# Test result communication in primary care: a survey of current practice

**DOI:** 10.1136/bmjqs-2014-003712

**Published:** 2015-08-04

**Authors:** Ian Litchfield, Louise Bentham, Richard Lilford, Richard J McManus, Ann Hill, Sheila Greenfield

**Affiliations:** 1 School of Health and Population Sciences, Medical and Dental Sciences, University of Birmingham, Birmingham, UK; 2 Health Sciences, Warwick Medical School, University of Warwick, Coventry, UK; 3 Nuffield Department of Primary Care Health Sciences, University of Oxford, Oxford, UK; 4 Department of Transformation, Worcestershire Acute Hospitals NHS Trust, Worcester, UK

**Keywords:** Primary care, Quality improvement, Surveys, Communication, Process mapping

## Abstract

**Background:**

The number of blood tests ordered in primary care continues to increase and the timely and appropriate communication of results remains essential. However, the testing and result communication process includes a number of participants in a variety of settings and is both complicated to manage and vulnerable to human error. In the UK, guidelines for the process are absent and research in this area is surprisingly scarce; so before we can begin to address potential areas of weakness there is a need to more precisely understand the strengths and weaknesses of current systems used by general practices and testing facilities.

**Methods:**

We conducted a telephone survey of practices across England to determine the methods of managing the testing and result communication process. In order to gain insight into the perspectives from staff at a large hospital laboratory we conducted paired interviews with senior managers, which we used to inform a service blueprint demonstrating the interaction between practices and laboratories and identifying potential sources of delay and failure.

**Results:**

Staff at 80% of practices reported that the default method for communicating normal results required patients to telephone the practice and 40% of practices required that patients also call for abnormal results. Over 80% had no fail-safe system for ensuring that results had been returned to the practice from laboratories; practices would otherwise only be aware that results were missing or delayed when patients requested results. Persistent sources of missing results were identified by laboratory staff and included sample handling, misidentification of samples and the inefficient system for collating and resending misdirected results.

**Conclusions:**

The success of the current system relies on patients both to retrieve results and in so doing alert staff to missing and delayed results. Practices appear slow to adopt available technological solutions despite their potential for reducing the impact of recurring errors in the handling of samples and the reporting of results. Our findings will inform our continuing work with patients and staff to develop, implement and evaluate improvements to existing systems of managing the testing and result communication process.

## Introduction

**Figure 1:**
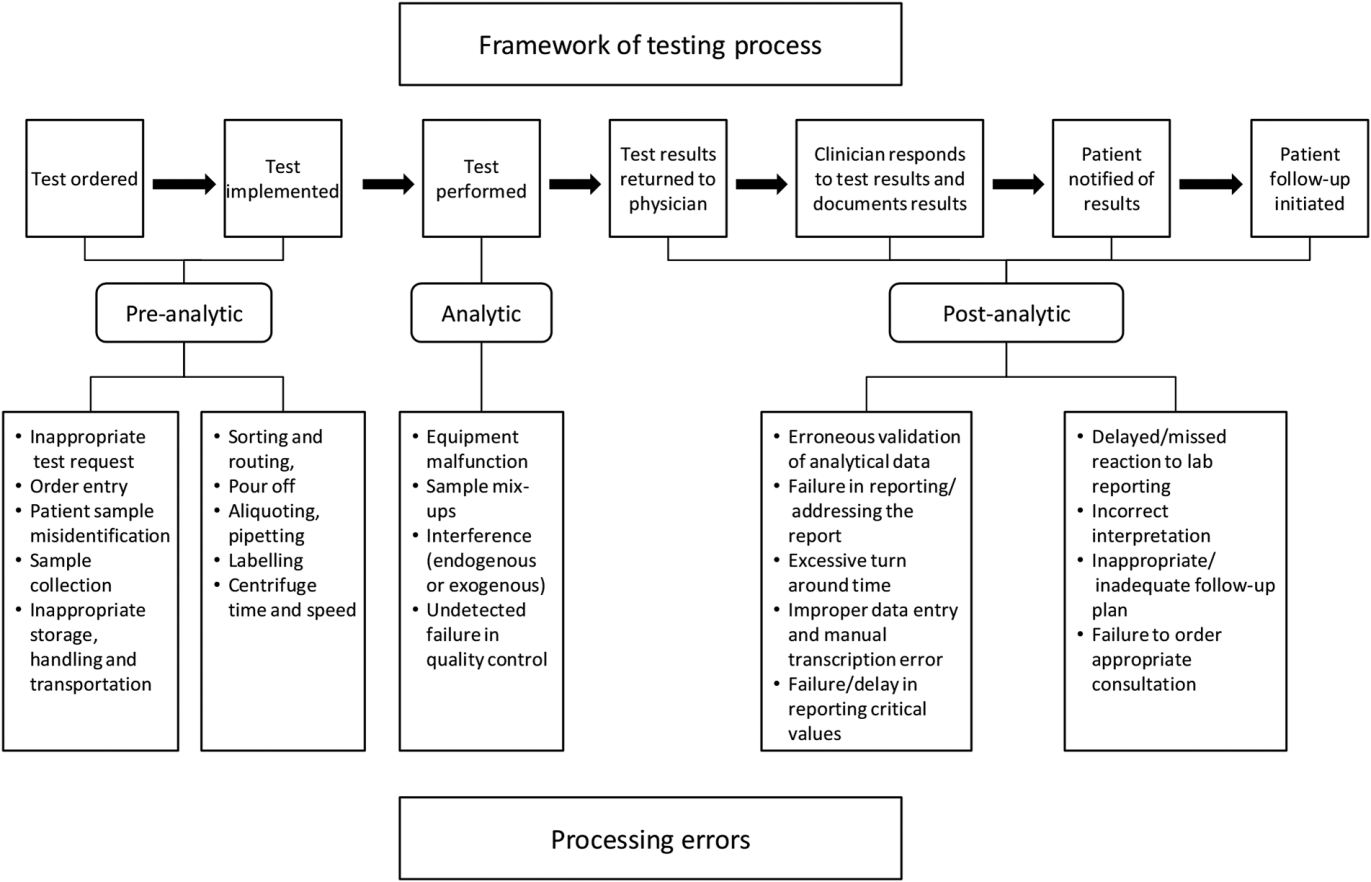
Conceptual framework for testing process and associated errors in sample processing after Hickner *et al*^2^ and Carraro and Plebani.^1^

A successful testing and result communication (TRC) process in primary care requires the coordinated efforts of general practitioners (GPs), patients, administrative personnel and laboratory staff performing a series of inter-related tasks (see figure 1).^1^ ^2^ Though the reasons for ordering tests may vary, the timely and accurate communication of results is central to ensuring the provision of appropriate care but is a complex and fragmentary process.^2^ As a consequence, there is an increased likelihood of mistakes from ordering and implementing tests^2^ ^3^ and handling samples^4^ to reporting results to clinical health professionals^2^ ^5^ ^6^ and notifying patients.^7^ ^8^ These errors surface within the primary care environment and laboratory settings, and previous studies have identified sampling errors and misidentification of samples as being among the most common.^1^ ^9–11^

The continued lack of systematic reporting of errors in primary care means it is likely that many remain undetected and the frequency of known errors underestimated,^12^ despite considerable implications for patient safety^3^ ^5^ ^7^ ^13^ ^14^ and medicolegal concerns for providers.^2^ ^15–17^

The need to address these issues in the UK is critical at a time when primary care is being asked to cope with increasing demand for tests from an ageing chronically ill population,^18^ combined with calls for improved patient access to their medical information.^19^ Surprisingly few studies have explored the issues around the TRC process in the UK.^20^ ^21^ Here we present the findings from two complementary pieces of work considering current UK practices for managing the TRC process from both practice and laboratory perspectives.

## Methodology

### Telephone survey of GP practices

#### Setting

We surveyed staff at a total of 50 general practices from across the 10 English Strategic Health Authorities (SHAs, disbanded on 1 April 2013 were replaced by Clinical Commissioning Groups and the NHS Trust Development Authority),^22^ purposively sampled to include a range of geographical regions, rural and urban locations, Index of Multiple Deprivation (IMD) codes,^23^ SHA and number of full-time and part-time equivalent GPs—see online supplementary table S1.

#### Design

Aware of the difficulties in engaging primary care staff,^24^ and of our need to cover a large geographical area,^25^ we conducted a telephone survey which consisted of partially categorised questions (see online supplementary table S2: General practice survey: Questions and categories). The content of the questions was informed by our previous focus group work with practice staff exploring TRC at four practices in the South Birmingham area.^26^

#### Participants

We surveyed either a practice manager (PM), lead receptionist or other suitable informant at each participating practice. Both groups of employees have responsibility for the effective running of practice information systems including compliance with data protection legislation. Their roles and responsibilities mean they are well placed to provide information on the current result communication processes.^27^ ^28^ Participants were recruited through a combination of emails and phone calls. In the event that practices were not contactable or refused to take part we contacted the next practice on the purposively sampled list until the prescribed number of practices had been interviewed. In order to gain the necessary 50 completed surveys we contacted 240 general practices from our list between January and October 2012. The characteristics of participating (vs) non-participating practices are included in online supplementary table S2 (see online supplementary material).

#### Data collection and analysis

The telephone survey was conducted by LB, a senior research nurse, and took 10–15 min, where appropriate multiple responses were recorded (please see online supplementary table S1: General practice survey: Questions and categories). We grouped the data into three themes: the return of results to practices from laboratories and to patients from practices (questions 1 through 4), responsibility for communicating results (questions 5 and 6) and the clinical management software (CMS) used by practices to manage the process (question 7). We produced simple descriptive statistics of all the data.^29^

### Paired interviews with laboratory staff

#### Setting

To gain a more comprehensive understanding of the links between practices and testing laboratories we undertook a series of three paired interviews between January and December 2012 with the same two senior staff members responsible for ensuring the successful handling and analysis of results at the Biochemistry and Endocrinology Laboratory situated within a large NHS hospital foundation trust.^30^ The laboratory was chosen as it served participating practices in the study and also because it was considered typical in terms of size and capacity in comparison to other NHS laboratories. It was subject to external accreditation by Clinical Pathology Accreditation (UK) to ISO15189 and provided a clinical biochemistry service to the NHS trust, local community and mental health trusts and 150 GP practices.

#### Participants

We used a purposive direct sampling strategy to select the key members of staff that were involved in the management of samples, analysis and communication systems.^31^ Using the principles of the key informant technique we selected representatives employed by the service of interest, who possessed both a broad knowledge of the relevant systems and work practices and a willingness to inform our work.^32^ A laboratory information system specialist was responsible for information technology and communication systems both in the laboratory, and critically with external partners. The business pathology manager was a trained clinical biochemist and held overall responsibility for sample management, analysis and issuing of results. We chose to conduct interviews together to allow the dynamics between these senior staff and their different yet complementary roles to validate and clarify the processes described and produce more complete data as interviewees fill in each other's gaps and memory lapses.^33^ The resulting data was used to produce a service blueprint that placed into context the corresponding viewpoints of practice and laboratory staff on testing and result management and highlighted potential sources of failure and delay.^34^ ^35^

#### Data collection and analysis

**Figure 2:**
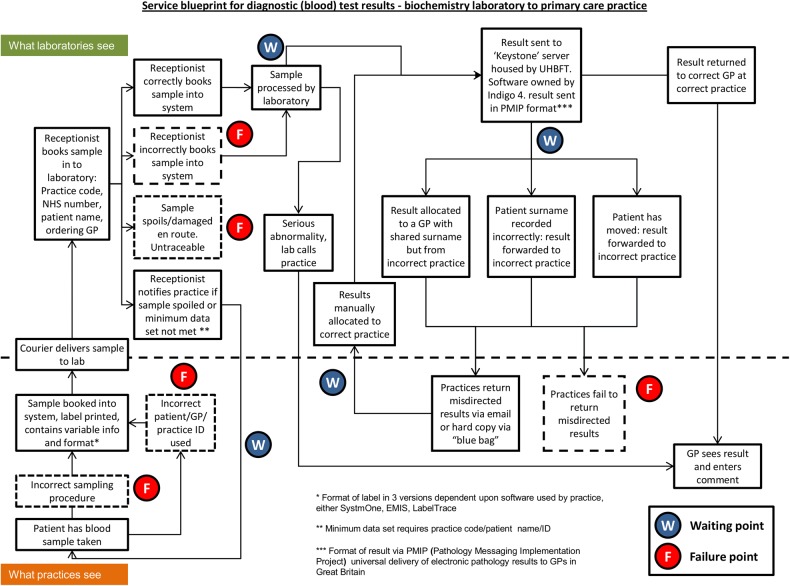
Service blueprint. EMIS, Egton Medical Information Systems; GP, general practitioner; PMIP, Pathology Messaging Implementation Programme; UHBFT, University Hospitals Birmingham NHS Foundation Trust.

The paired interviews were conducted at their shared office in the laboratory premises. Their responses were recorded as field notes by both the interviewer and a second member of the study team acting as an observer. A topic guide and prompts, informed by the previous focus groups and survey, were used to inform the discussions, which explored the processing of samples, communication with practices and potential sources of error (see online supplementary table S3: Topic guide for semistructured interviews with laboratory staff for topic guide). After the initial interview we outlined the underlying system and drafted a service blueprint which identified potential sources and locations of delays and error. We employed a deductive team-based approach to analysing the discussions and used them to inform the service blueprint.^36^ The updated draft blueprint was presented at each of the subsequent paired interviews until laboratory staff agreed that it offered an accurate portrayal of the process from the perspective of the managers interviewed. This iterative process produced the finalised blueprint shown in figure 2: a service blueprint for the links between a biochemistry laboratory and primary care practice for blood test results.

## Results

### Telephone interview survey

A total of 240 practices were contacted to take part in the survey. Practices were telephoned from a list of 240 practices consisting of 24 primary care trusts with 10 practices in each. Fifty practices completed the survey: 34 PMs, 4 deputy PMs, 10 lead receptionists, 1 lead nurse and 1 information technology (IT) lead. We received a positive response and arranged to interview 68 PMs who were subsequently unavailable to do the telephone survey at the agreed time. There was no answer to our telephone calls on either of two attempts at 73 practices. Forty one practices declined our invitation to take part saying either ‘we do not do surveys’ or ‘we are too busy’.

Participating practices reflected a range of IMD codes, size (by number of GP) and urban and rural classification codes. They were similar in these respects to non-participating practices (see online supplementary table S1).

### Return of test results

All practices in the survey reported that blood test results were typically returned either electronically (64%) using the Pathology Messaging Implementation Programme standard,^37^ or both electronically and by hard (printed) copy, posted to practices by the laboratory (36%). We asked practices about the default method for communicating normal results (where ‘normal’ is defined as requiring no further action) and 49 out of 50 practices (98%) required patients to contact the surgery (see online supplementary table S2). The exception was one ‘walk-in’ practice (2%) which sent text (SMS) messages to patients to advise that a normal test result had been returned and that no follow-up appointment was required.

The default communication method for abnormal results at 20 practices (40%) required patients to contact the practice as they would for normal results and only then would they be informed the results were abnormal. At 18 practices (36%) GPs telephoned patients if there was a sensitive or serious test result abnormality. At nine practices (18%) administrative staff telephoned patients with an abnormal result asking them to book an appointment. If they failed to reach the patient by telephone, a letter was issued asking them to contact the surgery. At two practices (4%) patients were required to telephone a receptionist to make a GP appointment to find out test results. The one ‘walk-in’ practice (2%) used SMS messages requesting patients to make an appointment for abnormal results (the results are summarised in online supplementary table S2). At 38 practices (76%) when the practice was unable to contact patients by telephone with abnormal results, administrative staff were allocated the task of writing to patients advising them that a GP appointment was needed.

When asked if the practice had any means of knowing if a blood test had been returned by the laboratory, five practices (10%) had an allocated staff member to check paper records of tests ordered against electronic patient records. Three PMs (6%) were confident their electronic record systems would highlight missing test results, though none were sure of the actual process or how they might locate this data. A further 42 practices (84%) had no system in place to detect whether a blood test had been returned by the laboratory. In each case the practice confirmed that they would only realise a result was missing if, following a patient call, the result could not be found on the system.

### Responsibility for communicating results

None of the practices surveyed had allocated responsibility to a specific team member to record whether abnormal results had been returned to patients.

### Clinical management software

A number of CMS systems are available in the UK. All have a degree of capability for facilitating the TRC process. In our sample either Egton Medical Information Systems or SystmOne, was used at 86% of practices. All of the systems had the functionality to return test results via SMS and the capacity to track and record tests ordered and results returned to practices.

### Testing and sample management: links between practice and laboratory

The senior laboratory staff described the TRC process and potential sources of delay (waiting points W1–4) and failure (failure points F1–5). The process was initiated when practices provided a hard copy of the order form and a sample labelled in one of several different formats depending on the software system employed by the practice. The laboratory provided a handbook for practices containing instructions on the minimum requirement of data for a sample to be accepted and analysed. Samples and accompanying request forms were collected by the NHS trust from each practice and delivered to the laboratory each weekday. The laboratory operated offline and so following delivery to laboratory reception, label details were manually entered into the system. Where a sample was spoiled or key data missing, the laboratory receptionist returned a null result to the ordering practice (waiting point, W1). Samples were typically analysed within 24 h though this timescale could be extended depending upon the test (W2). Electronic reports containing result data were issued every 24 h via a hospital server to practices which could issue a read receipt, though the absence of a receipt did not trigger further action by the laboratory.

Our discussions confirmed that mistakes in the sampling procedure or otherwise the mishandling of the sample by practice (failure point, F1) or laboratory staff (F3) could result in non-viable samples. Samples and results could also be lost or delayed due to identification errors occurring at both the practice (F2) and the laboratory where identification data may be incorrectly transcribed by staff (F4). These errors in sample identification could lead to delay (W1 and W3) and where label data is so imprecise or damaged as to render it untraceable, the sample may be lost (F5).

Following analysis, delay could occur if results were returned to the incorrect practice (W3). Where GPs were aware of a missing result, they could email the IT manager at the laboratory or call the laboratory directly when a computerised search could be undertaken. Where results were misallocated, practices returned them to the laboratory via email or as a hard copy, which is a lengthier process that could take weeks (W4). It is worth noting that not all results that arrive at GP surgeries in error are returned to the laboratory for redistribution (F5).

## Discussion

### Main findings

Here we present the first telephone survey of current TRC in primary care conducted in the UK. It has highlighted how current processes frequently depend on the actions of patients for the successful communication of normal and abnormal results and to identify results that are missing or delayed. The service blueprint arising from discussions with laboratory staff describes the interaction between laboratories and practices and locates persistent sources of error. Practices appear unaware and/or ill-equipped to detect such errors despite the existence of potential technological safeguards.

### Study limitations

The survey of general practices used partially categorised questions to aid the manual transcribing of responses. One of the major risks of this type of questions is that the respondent will precategorise too quickly, resulting in a potential loss of interesting and valuable information. In addition, interviewers may try to force the information into the listed categories instead of exploring the question more thoroughly.^38^ More detailed information on the result management process, such as the exact proportion of results returned to practices via hard copy or specific policies in place to cover absent GPs, was not collected. Funding limitations meant that only 50 practices could be included in the survey. Though this number is small for a survey of this type and the response rate was low, the similarities between the responses of those surveyed would indicate that we had captured the full range of responses. Furthermore, the range of clinical management systems used by our sample reflects the reported market share of each of the three systems.^39^ We acknowledge that surveying clinical staff such as practice nurses or GPs may have elicited different responses. The decision to survey senior administrative staff was based on their position within the practice that gave them an overview of the systems employed to manage samples.^28^ ^40^ However, 80% of practices either did not answer or refused to participate; this is not unusual when considering their workload^41^ and earlier low response rates in primary care.^24^

The limit in scope and size of our paired interviews means that we cannot pretend that our service blueprint is indicative of every laboratory that still operates offline in the UK. Interviews with small groups of staff are a valid source of data for a service blueprint,^33^ and the fact that many potential sources of error had been previously identified suggests that these are sound.^42^ ^43^

### The survey of practices

#### Reliance on patients for communicating results

The reliance on patient contact for retrieving normal, abnormal and missing results is a concern. Previous studies have shown that the clinical importance of the test is not a reliable indication of whether a patient will collect the result,^21^ and there is a growing body of evidence in a variety of settings that patients are failing to be notified about abnormal results.^13^ ^44^ ^45^ Recent work carried out as part of this study has found that frequently patients are unaware of their responsibilities in result communication.^46^ Only one of the practices we spoke to reported using SMS to proactively communicate results to patients and this was a walk-in centre.^47^ This asynchronous communication, which can also include access to results via web portals, texts or emails, can present its own problems.

Where used in the USA the mechanisms facilitating this interaction between patients and healthcare providers is not yet robust or integrated into current practice systems and there can be uncertainty for providers that the communication has reached the targeted individual.^48^ Despite this, early in 2014, direct and independent access to laboratory reports was approved in the USA.^49^ Meanwhile, in the UK initiatives are slowly being introduced to improve patient access to their healthcare information.^50^ Though none of the surveyed practices reported using email in result communication this may change when the current contract for secure communication in the NHS is renewed in spring of 2015 and will no longer include free SMS.^51^ With this in mind and with the proliferation of smart phones in the UK we may see providers increasingly use email in primary care result communication though this is dependent upon assuaging the concerns previously voiced by patients and providers about security and confidentiality.^52^

#### Tracking results

Practices did not have a specific member of staff whose role it was to ensure that abnormal results had been returned to patients. However, the clinical management systems employed by our sample carry the functionality to track both the return of results to the practice and whether they have reached patients. Previously in the USA, family practitioners have expressed dissatisfaction with the methods available for tracking abnormal results,^53^ though no such views were evident among the administrative staff we surveyed. Only four of the practices we spoke to reported using electronic methods to determine whether results had been returned to the practice from laboratories despite evidence indicating that a more consistent usage of software-based clinical management systems allied with the systematic labelling of samples can improve timeliness, recognise errors and reduce the frequency of missing results.^54^ ^55^ Previous work in the USA has looked at tracking results,^5^ ^56^ and it is worth noting that electronic handling does not necessarily mean a reduction in missing results.^57^ This process of adoption of new health information technologies is not straightforward and previous work has identified the complex technical and social factors that can influence the take-up of technological solutions.^53^ These range from the usability and functionality of the system,^58–60^ the characteristics of staff^61–63^ and the socioeconomic environment in which the practice resides.^64^

#### Improving management of current systems

Attempts have been made to improve result management in general practice and there is evidence that simple processes for managing results have been associated with reducing failure rates.^36^ ^44^ ^65^ More recently, researchers have recommended a systematic approach to improve the robustness of the TRC process, one that defines individual responsibilities and timescales to generate a workflow management model that would be most effectively employed alongside available technology designed for tracking tests.^66^

### The service blueprint: a laboratory perspective

We have outlined above the potential vulnerability of current systems from a primary care standpoint; however, this only tells part of the story. To provide context for the environment in which practices and patients negotiate the TRC, we need to understand the central role of laboratories and how they interact with practices in the UK. Though many of the issues we identified have been recognised previously, it is notable they persist, at least in the UK.^67^ ^68^ The service blueprint that emerged from our paired interviews with senior laboratory staff confirmed three predominant aspects of sample management that can lead to lost or delayed results: sampling and handling, identification and tracking.

Previously recognised errors in sampling such as incorrect sample volume and mishandling by general practice or laboratory staff can lead to non-viability of samples^12^ ^69^ and were identified as an ongoing problem. Successful strategies for reducing these types of error have been introduced in Spain;^42^ however, where they persist they risk introducing considerable delay for patients unless there is a fail-safe system to report and react to them.

Accurate information on the frequency of missing and delayed results is absent in the UK where the tracking of samples is hindered by the lack of a consistent system for labelling and identification. In Australia and the USA, a unique barcode for each patient as part of a fully electronic ordering system is being introduced.^12^ ^43^ ^54^ Combined with electronic ordering, the mutual tracking of samples has the potential to aid the prompt recognition of system failure^69^ and reduce delay from either repeating or redirecting tests. Such systems operate in some parts of the UK but the lack of a universal system hinders progress.

## Conclusion

Our study has shown that the potential for error in the TRC process is large yet seemingly unrecognised in general practice. Staff have yet to introduce technological solutions with the result that the robustness of the system hinges on patients who may be unaware of their responsibility to retrieve results and in many cases act as the system fail-safe. These issues are exacerbated by the persistent weakness in the management of samples and results by practices and laboratories. That many sources of error in the process have been recognised previously and that they remain unaddressed should be of considerable concern for both patients and providers.

A better understanding of the factors that contribute to the continued use of fallible systems is required and the input of all participants will be needed if sustainable improvement is to be introduced.^70^ ^71^ The results presented here will inform further research which needs to involve patients and staff to develop, implement and evaluate practicable, mutually agreed and much needed improvements to the process of TRC.

## Supplementary Material

Web supplement
