# Supplementary material for: Test result communication in primary care: a survey of current practice
Source: BMJ Qual Saf. 2015 Aug 4;24(11):691–9. doi: 10.1136/bmjqs-2014-003712 (PMC4680128; doi:10.1136/bmjqs-2014-003712)
Supplement: Web supplement [file bmjqs-2014-003712-s1.pdf]

SUPPLEMENTARY MATERIAL

Table 1: Characteristics of practices surveyed

Practice IMD Code	Participating practices	Non-participating practices	Number of GPs at practice	Participating practices	Non-participating practices	Urban/Rural Classification codes**	Participating practices	Non-participating practices
**1**	12	9	**1**	7	7	**A1**	17	17
**2**	6	10	**2**	8	4	**B1**	0	0
**3**	5	4	**3**	5	9	**C1**	27	18
**4**	11	2	**4**	13	8	**C2**	0	0
**5**	4	4	**5**	5	5	**D1**	6	4
**6**	1	1	**6**	5	4	**D2**	0	0
**7**	3	6	**7**	1	0	**E1**	0	1
**8**	2	1	**8**	3	2	**E2**	0	0
**9**	5	3	**9**	1	0	**F1**	0	1
**10**	1	1	**10+**	2	2	**F2**	0	0

* The IMD codes are produced by the UK government and first released in 2004 and updated in 2010. They provide indicators of deprivation in local authority areas to inform health and social policy. One is more deprived and ten less deprived,[47].

** Source:https://www.gov.uk/government/publications/2011-rural-urban-classification: Urban: Major Conurbation(A1), Urban: Minor Conurbation (B1), Urban: City and Town (C1), Urban: City and Town in a Sparse Setting (C2), Rural: Town and Fringe (D1), Rural: Town and Fringe in a Sparse Setting (D2), Rural: Village (E1), Rural: Village in a Sparse Setting (E2), Rural: Hamlets and Isolated Dwellings (F1), Rural: Hamlets and Isolated Dwellings in a Sparse Setting (F2)

Table 2: General practice survey: Questions and categories

<table>
<tr><th colspan="2">Questions</th><th>Categories</th></tr>
<tr><td>1.</td><td>How are test results returned to the practice by laboratories?</td><td>• PMIP
• Hard Copy</td></tr>
<tr><td>2.</td><td>How do patients typically receive normal results?
(select one)</td><td rowspan="2">• Ordering GP calls with results
• Triage GP calls with results
• Practice nurse calls with results
• Receptionist calls with results
• Task of phoning not allocated to a specific person
• Text message sent to patient
• Email sent to patient
• Letter sent to patient
• Patient phones practice
• Patient accesses shared electronic record
• Patient makes appointment to see GP
• Patient visits practice and speaks to receptionist
• Other</td></tr>
<tr><td>3.</td><td>How do patients typically receive abnormal results?
(select one)</td></tr>
<tr><td>4.</td><td>How do you know if a test result has not been returned to the practice?
(select one)</td><td>• No system in place
• Responsibility assigned to a member of staff
• Alert generated by clinical management software
• Unsure</td></tr>
<tr><td>5.</td><td>Who is responsible for recording that normal results have been successfully delivered to patients?
(select more than one response)</td><td rowspan="2">• Patient's GP
• Triage GP
• Designated nurse
• Receptionist
• None allocated</td></tr>
<tr><td>6.</td><td>Who is responsible for recording that abnormal results have been successfully delivered to patients?
(select more than one response)</td></tr>
<tr><td>7.</td><td>What electronic patient management software is used by the practice?
(select one)</td><td>• EMIS versions
• System One
• Vision</td></tr>
</table>

Table 3: Topic guide for paired interviews with laboratory staff

Questions		Prompts
1.	Processing of sample	Labelling of sample<br>Delivery of sample<br>Time taken to process sample<br>Laboratory interaction with practice software
2.	Communication with practices	The use and prevalence of PMIP<br>Clinical software systems<br>Checks on results<br>Methods of reporting results
3.	Potential sources of error	Sample handling<br>Mislabelling<br>Failure to return results
